# Personal

**Published:** 1905-11

**Authors:** 


### PERSONAL.

Dr. ARTHUR R. REYNOLDS, formerly commissioner of health at the university, and also the office of chief of staff at the hos- of Chicago, announces that he has accepted the position of medical director of the French Lick Springs Hotel Company at Fhench Lick, Indiana. The directions of physicians relative to their patients will be strictly observed, and correspondence is invited.

---

Dr. J. H. CARSTENS, of Detroit, was elected president of the Mississippi Valley Medical Association at its recent meeting at Indianapolis. The next meeting will be held at Little Rock.

---

Dr. MATTHEW D. MANN, of Buffalo, was one of the guests of honor at the annual meeting and dinner of the Ccicago Gyneological Society, held October 20, 1905.

---

Dr. MARCUS ROSENWASSER, of Cleveland, was the guest of honor at a dinner tendered him by fifty physicians, October5, 1905. Dr. Rosenwasser, who is professor of gynecology in the Cleveland College of Physicians and Surgeons, has recently spent a year in Europe, and his colleagues signalled his return in the manner mentioned.

---

Dr. WILLIAM H. SAUNDERS, of Montgomery, Ala., is appointed to deliver the oration on "State Medicine" at the annual meeting of the American Medical Association, to be held at Boseon next June.

---

Dr. ALLEN A. JONES, of Buffalo, was elected president of the New York State Medical Association for the ensuing year, at its recent annual meeting held at New York.

---

Dr. WILLIAM D. HAGGARD, of Nashville, delivered the address on surgery, at the recent meeting of the Mississippi Valley Medical Association, held at Indianapolis, choosing for his subject The Present Status of the Surgery of the Stomach.

---

Dr. GEORGE BEN JOHNSTON, of Richmond, professor of gynecology and abdominal surgery in the Medical College of Virginia, has been tendered, by the trustees of the University of Virginia at Charlottesville, the chair of professor of surgery pital. Dr Johnston's acceptance of these chairs is announced in recent issues of the Richmond papers, to take effect with the beginning of the session of 1906.

---

Dr. NICHOLAS SENN, of Chicago, was tendered a dinner by his professioned colleagues, Saturday, November 11, 1905, at the Auditorium. The *Medical Standard* gave considerable space in its November issue to an account of Professor Senn's surgical work. A full-page portrait of the distinguished surgeon was published as a frontispiece.

The following program was observed: presentation of medallion, Dr Joseph D. Bryant, president of the medical society of the state of New York; presentation of loving cup on behalf of Professor Senn's former students, Dr. Lewis G. Nolte, Milwaukee—response by Dr. Senn; "American Surgery," Dr. William J. Mayo, president-elect of the American Medical Association, Rochester, Minn.; "The American Medical Association," by its president, Dr. Lewis S. McMurtry, Louisville; "The Medical Man as the Patient sees him,"Hon. George R. Peck, Chicago; "The Medical Man vs. the Surgeon," Dr. John A. Witherspoon, Nashville; "American Medical Literature," Dr. Charles A. L. Reed, Cincinnati; short reminiscences, anecdotes, etc. by Dr. Jasob Lang, Milwaukee; Edward Boeckmann Saint Paul; Fernand Henrotin, Daniel R. Brower, William E. Quine, Chicago; and others. The guests, to the number of 850 assembled at the Fine Arts entrance to the Auditorium at 6.30, and it was midnight when the banquet was ended.

---

Dr. JOHN B. MURPHY, of Chicago, gave a breakfast at 12 o'clock the next morning after the banquet to professor Senn, which latter is described elsewhere. Dr. Murphy had as his guests,—besides Dr. Senn,—Drs. Ochsner, Webster, Bevan, Billings, and Quine, Chicago; Bryant, New York; Witherspoon, Nashville; W. J. Mayo, Rochester, Minn.; Reed, Cincinnati, and McMurtry, Louisville. It was a rare occassion.

---

Dr. D. W. TOTMAN, of Syracuse, was elected president of the Medical Association of Central New York, at its recent meeting held at Buffalo.

---

Dr. A. L. BENEDICT, of Buffalo, read a paper on Determination of the Gastric Area, with special reference to hourglass stomach, transportation of viscera, gastroptosis, etc., before the Pittsburg Academy of Medicine, Tuesday evening, October 10, 1905.

